# Gender disparities in the adoption of improved management practices for soybean cultivation in North East Nigeria

**DOI:** 10.1016/j.jafr.2025.102032

**Published:** 2025-08

**Authors:** Amadu Yaya Kamara, Lucy Sahbong Kamsang, Amina Mustapha, Alpha Yaya Kamara, Adetomiwa Kolapo, Nkeki Kamai

**Affiliations:** a Department of Agricultural Economics and Extension, Obafemi Awolowo University, Ife, Nigeria; b Department of Agricultural Economics and Extension Ahmadu Bello University, Zaria, Nigeria; c Department of Agricultural Economics and Extension Bayero University, Kano, Nigeria; d International Institute of Tropical Agriculture, Kano, Nigeria; e Department of Crop Science, University of Maiduguri, Nigeria

**Keywords:** Soybean, Adoption, Gender, Improved soybean management practices, Borno state, Nigeria

## Abstract

This study examined gender disparities in the adoption and intensity of improved soybean management practices among 800 farming households in Borno State, Nigeria, with equal representation of male-led and female-led households. The findings reveal that while both male and female farmers adopt improved soybean varieties, fertilizer, and herbicides, the intensity of adoption varies due to differences in socioeconomic constraints. Male farmers demonstrated slightly higher adoption rates across all practices, particularly for herbicide use. However, financial and market-related barriers, such as high input costs and distance to seed markets, disproportionately hindered female farmers' ability to fully integrate improved practices into their farming activities. The analysis indicates that the adoption of improved soybean varieties by male farmers was mainly influenced by income and pest/disease constraints, whereas female farmers were more affected by age, extension visits, and community tenure. Herbicide use among male farmers was driven by farm size and input costs, while for female farmers, it was influenced by education, input costs, and proximity to seed markets. Fertilizer adoption among male farmers was linked to income and farming experience, whereas female farmers' fertilizer use was shaped by farm size and financial constraints. Ordered probit regression results suggest that age negatively affects adoption intensity for both genders, but income and community tenure play a stronger role for men, while market access and cost barriers are more significant for women. Improving access to extension services can significantly enhance adoption rates, especially for female farmers who face higher input costs and limited access to seed markets. Targeted subsidies and credit programs tailored to smallholder farmers will help alleviate financial barriers, enabling both men and women to invest in essential inputs and expand production. Strengthening rural infrastructure, including better road networks and input market accessibility, will further reduce logistical challenges and support increased soybean cultivation.

## Highlights

• An estimated 75.25 % of male farmers and 69.5 % of female farmers adopted improved soybean varieties, showing strong uptake across genders.
• Fertilizer and herbicide use were nearly equal for both genders, with 68.5 % of males and 68 % of females using fertilizers; herbicide use was slightly higher among males (16.5 %) than females (14.5 %).
• Approximately 12.63 % of male farmers did not adopt any improved soybean management practices (ISMPs), with slightly higher non-adoption among females (13.75 %) compared to males (11.5 %).
• Influencing factors differed by gender: male adoption was driven by income and pest constraints, while age, extension visits, and community tenure were more significant for female adoption.
• Gender mainstreaming efforts in soybean interventions were effective, with high and equitable adoption rates and support accessible to both genders.

## Graphical abstract

**Figure:**
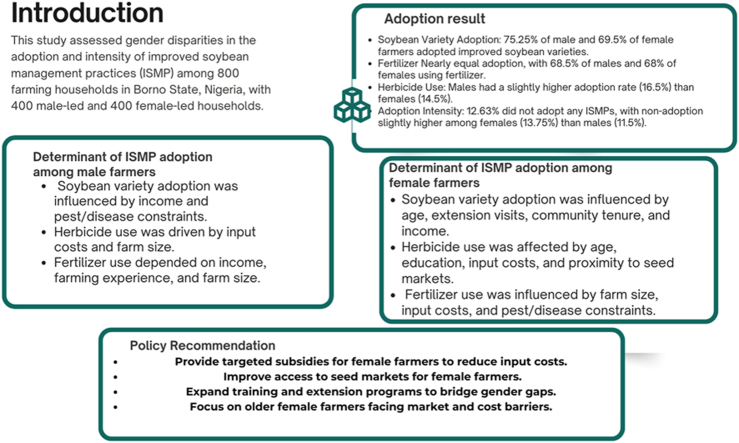

## 1 Introduction

**Fig. 1:**
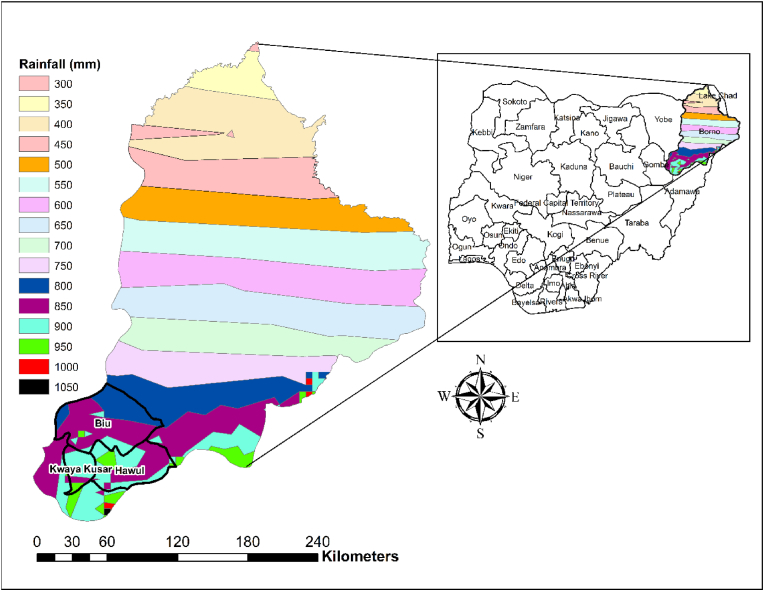
Map of study area.

Soybean (*Glycine max*.) is a legume crop in northern Nigeria of great importance, due to its potential to generate income for rural farmers, contribution to soil fertility improvement, and its use in traditional dishes [1,2]. Such is the importance of soybean that in 2018, Nigeria produced approximately 758,033 tons of soybean over 780,679 ha, ranking second in Africa after South Africa [2]. In Borno State and other parts of North East Nigeria, soybean cultivation holds particular importance for women, a contrast to traditional cereals like sorghum or maize, where female participation is limited [2]. Women not only participate in growing the crop but also play significant roles in processing and marketing soybean products, which substantially boosts their economic prospects along the value chain [3]. However, challenges for female farmers have been intensified by the Boko Haram insurgency, which has resulted in significant displacement and restricted access to essential farming resources [4]. These challenges are worse for female farmers who are already constrained by access to inadequate labor and limited financial resources [2,5]. Additionally, systemic gender inequality influences the agricultural extension services available to farmers, where cultural and religious norms frequently obstruct effective interactions between male extension agents and female farmers [2]. These gender disparities in technology adoption efforts consequently impact agricultural productivity, income generation, and food security, ultimately influencing long-term adoption rates [6] This challenge is particularly evident in Sub-Saharan Africa (SSA), where institutional frameworks often favor men, limiting women's access to education, extension services, and critical agricultural information [7] As a result, female farmers encounter greater social and economic barriers, restricting their ability to adopt and benefit from improved agricultural technologies at the same rate as their male counterparts (see Fig. 1).

Because soybean is a cash crop the opportunities to transform the quality of life of women is vast, even in the presence of these enormous challenges [8]. For example, in the food industry, soybean is transformed to cooking oil and processed into animal feeds [9]). The crop also enhances soil fertility through biological nitrogen fixation and acts as a trap crop for controlling the parasitic weed *Striga hermonthica* when rotated with cereals, to greatly improve the legume-cereal cropping system [10,11].

However, biotic and abiotic constraints have reduced the average soybean yield to less than 1 tonha^−1^, far below the potential yield of over 3 tons ha^−1^ [12]. Factors such as pests, diseases, drought, poor soil fertility, high pod shattering, and inadequate agronomic practices contribute to these low yields [13]. To address these challenges, the International Institute of Tropical Agriculture (IITA), along with its partners, has developed soybean varieties tailored for cultivation in the Nigeria savannas [14]. These varieties are resistant to diseases and drought and exhibit minimal pod-shattering [15]. IITA has promoted the adoption of these varieties in northeast Nigeria alongside essential agronomic practices, including the use of inorganic fertilizers and herbicides through projects like the Promoting Sustainable Agriculture in Borno State (PROSAB) [16]. The TLII (2007–2015) and N2Africa (2014–2018) projects funded by BMGF were also launched in the same region to increase legume productivity through increased biological nitrogen fixation and legume consumption to improve nutrition [17]. Before 2008, the PROSAB project largely introduced and promoted the soybean variety, TGX 1448-12E over traditional varieties grown in the region. However, TGX 1448-12E, is late maturing and relatively low yielding due to its susceptibility to soybean rust disease and delayed flowering as a result of photosensitivity. Because of these limitations, the TL-11 and N2Africa projects supported the dissemination of new varieties to address the constraints [18]. The new varieties introduced are early-maturing varieties, TGX 1951-3F, TGX 1955-4F and TGX 1904-6F, and an extra-early maturing variety, TGX 1835–10F, which are high yielding, drought tolerant and resistant to pests and disease [19]. The projects identified community-based organizations and worked with lead farmers to disseminate these soybean varieties through field demonstrations, field days and the mass media [17]. These projects all incorporated gender-mainstreaming activities, to ensure equitable participation in the technologies that they introduce [2]. To fully maximize the genetic potential of these yields IITA has also promoted complementary agronomic practices that are essential in creating the right environment for the varieties to strive. These include the promotion of fertilizers and the promotion of herbicides. Effective weed control through herbicides and the targeted application of inorganic fertilizers replenishes essential nutrients in the nutrient-deficient soils of the region [13].

Despite these efforts, detailed studies in Nigeria evaluating the combined adoption of these technologies, particularly for soybeans, are scarce. Thus the study seeks to assess the adoption levels of these agronomic practices and the factors influencing both their level and intensity of adoption. This is crucial as individual adoption of these practices is not sufficient; farmers must adopt them in combination to achieve significant improvements in their yield. While gender disparities in the adoption of individual agricultural technologies are well-documented, there remains a notable gap in the literature on gender differences in the adoption of multiple complementary technologies. This study aims to address this gap by providing empirical insights into how men and women adopt integrated agricultural management practices, contributing to a more comprehensive understanding of gendered technology adoption dynamics. Identifying the barriers to spreading improved agronomic practices allows for designing targeted interventions to encourage their uptake [20]. Considering the pivotal role of soybean for women's welfare in Northern Nigeria—a region where religious and ethnic barriers often restrict women's agricultural involvement—this study also explores gender differences in the adoption rates. It aims to identify specific factors influencing the adoption and intensity of improved agronomic practices among male and female farmers separately. This focus is crucial given the broader context of gender inequality, which not only hinders rural development but also exacerbates food insecurity by limiting women's access to resources like land, labor, education, extension services, and credit [21].

. While previous interventions, such as those led by IITA and its partners, have focused on technology dissemination and project implementation, this study takes an independent approach by assessing the adoption levels and intensity of improved agronomic practices among male and female farmers. It examines the factors influencing adoption beyond project settings, providing a data-driven analysis rather than an evaluation of specific interventions.

Theoretical Framework: Understanding Gender Disparities in Technology Adoption.

A comprehensive theoretical framework for analyzing gender disparities in technology adoption requires examining the social, economic, cultural, and institutional factors that influence the adoption of new technologies by men and women. To situate this study within a robust theoretical context, we integrate key frameworks that explain variations in adoption behavior: the technology acceptance model (TAM), gender role theory, social cognitive theory, and diffusion of innovations theory. These theories provide insights into the psychological, sociocultural, and structural barriers that impact technology adoption among different genders.

The Technology Acceptance Model suggests that an individual's decision to adopt technology is primarily driven by perceived usefulness and perceived ease of use [22]. Research has shown that gender differences exist in technology adoption due to variations in self-efficacy and perceived ease of use, with women often exhibiting lower confidence in adopting new technologies [23] For instance, studies in educational technology and e-banking found that women tend to rely more on external factors like training and institutional support for adoption, whereas men prioritize usefulness and autonomy in decision-making [23]. In agricultural contexts, women may perceive certain innovations as less beneficial or harder to implement, particularly when extension services are not tailored to their needs. Addressing this issue requires gender-sensitive training programs that consider women's unique challenges in accessing and implementing new technologies.

Gender Role Theory explains how socially constructed norms and expectations influence behavior, shaping technology access and decision-making [24]. Research highlights that traditional gender roles restrict women's participation in technology adoption due to limited access to financial resources, training programs, and decision-making power [25]. In agricultural societies, where men are often the primary decision-makers, women face structural barriers that limit their control over technology and financial capital []. Programs promoting joint household decision-making and gender-inclusive policies can help address these disparities and facilitate more equitable technology adoption.

Social Cognitive Theory (SCT) highlights the role of self-efficacy and observational learning in shaping technology adoption behaviors [26]. [26]Studies indicate that women often have lower self-efficacy in using technology due to limited exposure to role models and societal discouragement [26]. Research also suggests that women's confidence in technology adoption increases when they receive targeted training, mentorship, and opportunities for hands-on experience [27]. Therefore, interventions aimed at increasing women's self-efficacy—such as female-led extension programs and peer-to-peer learning networks—can significantly enhance their technology adoption rates.

The Diffusion of Innovations Theory explains how new technologies spread within a population, emphasizing factors like compatibility, complexity, observability, and trialability [28]. Gender disparities emerge when women have fewer opportunities to observe or experiment with new technologies, particularly in male-dominated industries [22,28] Studies have shown that innovations often spread through social networks where women are underrepresented, limiting their access to information and peer support [29]. Designing agricultural technologies that align with women's existing roles and ensuring their participation in farmer cooperatives can facilitate greater adoption.

Taken together, these theories illustrate that gender disparities in technology adoption result from a combination of individual, societal, and institutional factors. Addressing these disparities requires a holistic approach that tackles both structural and behavioral barriers to technology adoption.

One crucial step is challenging restrictive gender norms through community engagement and policy advocacy. Traditional gender roles often limit women's participation in technology adoption, particularly in agricultural settings where men are typically seen as decision-makers. By fostering awareness and implementing policies that promote gender equality, communities can create an environment where women have greater autonomy and access to technological advancements [25].

Another key aspect is enhancing self-efficacy among women through tailored training programs and female-led extension services. Many women lack confidence in using new agricultural technologies due to limited exposure and societal discouragement. Providing targeted training, mentorship programs, and female extension workers can empower women with the skills and knowledge necessary to adopt and effectively use technology [26].

Additionally, agricultural technologies must be designed to consider the specific needs and constraints of female farmers. Women often face unique challenges, such as time constraints due to household responsibilities and limited access to financial resources. Developing gender-responsive technologies that align with women's agricultural roles and ensuring their accessibility can significantly improve adoption rates [22].

Finally, ensuring women's participation in farmer networks, credit programs, and leadership roles is essential for sustainable and widespread technology adoption. Women frequently have limited access to these critical resources, which hinders their ability to invest in and implement new technologies. By promoting inclusive policies and creating opportunities for women to take on leadership positions within agricultural cooperatives and financial programs, their long-term participation in technological advancements can be secured [29].

## 2 Methodology

### 2.1 Study area and sampling

The study covered Biu and Hawul Local Government Areas (LGA) in the northern Guinea savanna and Kwaya Kusar LGA in the Sudan savanna zone in Borno State of northeast Nigeria. Sex-disaggregated sampling framework was used for the survey of male and female farmers. A multi-stage sampling approach was used (see Table 1 below). Firstly, three LGAs were purposefully selected in the Southern part of the State. These LGAs fall within the intervention sites of the past PROSAB, TLII and N2Africa project areas and cover the major soybean producing areas. In the second stage, a probability proportional to size sampling was used to randomly select fourteen communities from the list of communities in Hawul and Biu LGAs and twelve communities from Kwaya Kusar, which gives a total sample of forty communities. In the third stage, a sampling frame of households was constructed for the 40 communities [2]. In each community, twenty farming household heads (10 males and 10 females) were randomly selected from a list of all the farmers in the community, resulting in 400 male-headed households and 400 female headed-households giving a total sample of 800 households [2]. In constructing the sampling frame for female-headed households, we deliberately included only divorced, separated and widowed women. This decision reflects the sociocultural context of northern Nigeria, where, due to entrenched patriarchal norms, women typically assume the role of household head only in the absence of a male partner, such as through divorce or widowhood (see Table 2).

**Table 1:** Sampling technique.

LGAs	Stage 1: Purposive selection of LGAs	Stage 2: Probability proportional to size sampling to select communities	Stage 3:Simple ramdom sampling to select of households
Hawul	Selected	14 communities	280 households (140 M, 140 F)
Biu	Selected	14 communities	280 households (140 M, 140 F)
Kwaya Kusar	Selected	12 communities	240 households (120 M, 120 F)
Total	3 LGAs	40 communities	800 households (400 M, 400 F)

**Table 2:** Description of explanatory variables used in the empirical model.

Variable	Description
Age	Age
Education of HH head	Years of education of household head
Household size	Total members in farmer's household
Farm Experience	Total years spent as independent farmer
Membership of association	Membership of association 1 = yes 0 = otherwise
Frequency of extension contact	Umber of extension visit received in last 3 months
Total credit	Total credit received per household (Naira)
Income	Total income received per household (Naira)
Assets	Total value of household assets per capital (naira)
Tropical Livestock Unit size	Tropical Livestock Units are livestock numbers converted to a common unit. Conversion factors are: cattle = 0.7, sheep = 0.1, goats = 0.1, pigs = 0.2, chicken = 0.01.
Total land cultivated	Total land cultivated in 2016 measured in hectares
Use of SSP	Did farmer use SSP fertilizer 1 = yes 0 = otherwise
Use of herbicide	Did farmer use herbicides 1 = yes 0 = otherwise
Crop input cost	Total expenditure on seed, fertilizer, pesticide, labour per hectare (measured in Naira)
Constrained by low soil fertility	Perceived severity of constraints on a scale of 10, from zero (not constrained) to 10 (severely constrained),
Constrained by high cost of inputs	
Constrained by pests and diseases	
High oil content	Did variety have a high oil content 1 = yes 0 = otherwise
High fodder yield	Did variety have a high fodder yield 1 = yes 0 = otherwise
Less shattering	Did variety have less pod shattering 1 = yes 0 = otherwise
Distance to seed market	Distance to seed market (km)
Distance to extension service	Distance to extension service (km)
Distance to chemical market	Distance to chemical market (km)
Biu LGA	Resident in Biu LGA
Kwaya Kusar LGA	Resident in Kwaya Kusar LGA

The survey was implemented in October–November 2017 using survey questionnaires prepared and administered by trained enumerators who collected data from households through personal interviews with the help of computer-assisted personal interviews using the open data kit (ODK) software. Data were collected on the farmers’ socio-economic characteristics which include sex, education, age, farming experience, land ownership, social capital, access to extension and credit, and agronomic practices as well as varieties planted, major constraints they face in production of improved soybean varieties. Adoption of improved Soybean Varieties" pertains to the deliberate and continuous engagement by agricultural households in the cultivation of improved soybean varieties. Specifically, for the context of the 2017 cropping season, this includes households that have integrated any of the following early and extra-early Improved Soybean Varieties (ISVs): TGX1904-6F, TGX1835-10F, TGX 1951-3F, and TGX 1955-4F, into their farming operations across one or more agricultural plots.

Herbicide use refers to the application of chemical substances designed to control, suppress, or kill unwanted plant species in soybean cultivation. Adoption of herbicide use will be gauged by the percentage of farmers incorporating herbicides into their agricultural practices. Fertilizer use in soybean cultivation involves the application of inorganic materials particularly phosphorous which is applied in the form of Single Super Phosphate (SSP). Thus, the rate of adoption of fertilizer use can be assessed by the extent to which farmers use recommended types and quantities of fertilizers.

### 2.2 Econometric framework

#### 2.2.1 Multivariate probit analysis for soybean cultivation practices

Soybean farmers have the choice of a range of improved soybean management practices (ISMPs) to boost their productivity. To understand the factors influencing the adoption of these ISMPs, we hypothesized a connection in the error terms of these separate practices, comprising fertilizer usage (F), incorporation of improved seeds (S), and herbicide application (H). To investigate this, we selected the Multivariate Probit (MVP) due to its capacity to simultaneously evaluate the determinants of the collective adoption of ISMPs by soybean farmers. This simultaneous approach helps to uncover the relationship in error terms, which can indicate whether the practices are complementary (positive sign) or substitutive (negative sign). In adherence to the utility maximization framework, soybean farmers in North East Nigeria will adopt ISMPs if they foresee the benefits outweighing the non-adoption costs, such that if $U_{f}$ represent the utility gained by adopting the jth agronomic practice and $U_{n}$ represent the contrary [30] That is to say farmers will embrace the $j^{th}$ agronomic practice if $Y_{ij}=U_{f}–\;U_{n}>\;0\text{.}$

This framework can be represented as a binary outcome for every ISMP decision made by these farmers:

$$Y_{ij}=\left\{ \;\begin{array}{c} {1\;if\;Y}_{ij}>0\\ \;0\;if\;otherwise \end{array}\right.$$

where j=F,S,H

This utility framework can be expressed in the following model:

(1) $$Y_{\text{ij}}=\beta_{j}X_{i}+\varepsilon_{i}$$

Where $X_{i}$ stands for an array of independent variables, j symbolizes the soybean cultivation practice, $\beta_{j}$ denotes the parametric coefficient and $\varepsilon_{i}$ represents the error term.

Thus the probability of adoption of the ith farmer is given by Ref. [31] in Eq. (2):

Prob(Y=1∣x)=F(x,β)

(2) Prob(Y=0∣x)=1−F(x,β)

Where $F\left(x,\beta \right)=x^{\prime }\beta$

The functional form of Eq (1) depends on the assumption made for the error term ϵ, which is assumed to be normally distributed in a probit model. Thus, for the *i*th farmer, the probability of the adoption of ISMPs practices is given by:

(3) $$Prob\left(Y=1|x\right)=\int -\infty x^{\prime }\beta \varphi \left(t\right)dt=\Phi \left(x^{\prime }\beta \right)$$

where Φ(t) is the cumulative distribution function of the standard normal. Since we are interested in accommodating multiple soybean production practices, we adopt the multivariate probit model with multiple equations that is based on the same principle. The resultant equation system is given by Ref. [31]:

(4) $$y_{m}*=x_{m}^{\prime }\beta \;m+\epsilon_{m}\;y_{m}=1\;\text{if}\;y_{m}*>0,0\;\text{otherwise},m=1,\ldots ,M$$

This equation has the following properties according to Rahman & Chima [30]:

The expected value (mean) of the error term $\epsilon_{m}$ given the covariates $x_{1},\ldots ,x_{M}$ is zero. This is a standard assumption in regression models, implying that the errors have no systematic bias.

$$E\left[\epsilon_{m}|x_{1},\ldots ,x_{M}\right]=0$$

the variance of the error term $\epsilon_{m}$ given the covariates is equal to one. In the context of the multivariate probit model, this normalization helps in identifying the model parameters since the scale of the latent variable is not identifiable.

$$\text{Var}\left[\epsilon_{m}|x_{1},\ldots ,x_{M}\right]=1$$

The covariance between error terms $\epsilon_{j}$ and $\epsilon_{m}$ given the covariates is $\rho_{jm}$. The $\rho_{jm}$ terms represent the correlation between the unobserved factors affecting the binary outcomes. This correlation allows the model to capture dependencies between different binary outcomes.

$$\text{Cov}\left[\epsilon_{j},\epsilon_{m}{|}_{x}1,\ldots ,x_{M}\right]=\rho_{jm}$$

the vector of error terms ($\epsilon_{1},\ldots ,\epsilon_{M}$) follows a multivariate normal distribution with mean vector 0 and covariance matrix R. The covariance matrix R captures the covariances $\rho_{jm}$ between the error terms of different equations, encapsulating the correlation structure of the multivariate probit model.

$$\left(\epsilon_{1},\ldots ,\epsilon_{M}\right)∼NM\left[0,R\right]$$

Presuming a simultaneous adoption of ISMPs, the error terms for this equation can be illustrated using a variance-covariance matrix as in Eq. (5):

(5) $$\epsilon_{j}=\lceil \begin{array}{ccc} 1 & \;\delta \text{FS}\; & \;\delta \text{FH}\\ \;\delta \text{SF}\; & 1 & \;\delta \text{SH}\;\\ \delta \text{HF}\; & \;\delta \text{HS}\; & 1 \end{array}\rceil$$

where rho (δ) is the correlation between two distinct ISMPs. The δ sign between any two practices denotes their interrelation, with a positive sign symbolizing complementarity and a negative one indicating substitution.

#### 2.2.2 Ordered probit model

The ordered probit model is employed in this study to measure the intensity of adoption of improved soybean management practices (ISMPs), specifically focusing on Fertilizer Usage (F), Incorporation of Improved Seeds (S), and Herbicide Application (H) in soybean cultivation in North East Nigeria. The intensity of ISMP adoption is defined as the number of ISMPs that a household adopts. Similar to previous studies, due to data limitations, we treated our regressand used to measure adoption intensity as an ordinal variable with categories of ordered outcomes ranging from one to four, depending on the number of ISMPs a household adopted. The ordered probit is used because the intensity of ISMP adoption variable is both discrete and ordinal.

The ordered probit on a latent random variable as applied by Kolapo et al. [32]on and Trivedi [33] is:

(5) $$y_{i}^{*}=x_{i}^{\prime }\alpha +e_{i,}\;i=1,2,\ldots ,N$$

Where $E\;\left(e_{i}|x_{i}\right)=0$ and $Var\left(e_{i}|x_{i}\right)=1$. When the observed variable $y_{i\;}$, is a categorical variable with k response categories we can use it as a proxy for the latent (unobserved) random variable $y_{i}^{*}$. In the presence of a vector of unobservable threshold parameters ($\pi =\pi_{-1}\pi_{0}\pi_{1}\ldots \pi_{k-1}\pi_{k}$, the relationship between the observed and latent variables can be written as

(6) $$y_{i\;}=k\;\text{if}\;\pi_{k-1}<y_{i}^{*}\leq \pi_{k},k=0,1,2,\ldots ,K$$

Where $\pi_{k-1}=-\infty \text{,}$ $\pi_{0}=0\text{,}$ $\pi_{k}=\infty$ and $\pi_{-1}<\pi_{0}<\pi_{1}<\ldots <\pi_{k}$. The probabilities will thus be given as follows:

(7) $$Prob\;\left[y_{i\;}=k\right]=Prob\left[\pi_{k-1}<y_{i}^{*}\leq \pi_{k}\right]=Prob\left[\pi_{k-1}-x_{i}^{\prime }\alpha <e_{i,}\leq \pi_{k}-x_{i}^{\prime }\alpha \right]=\varphi \left(\pi_{k}-x_{i}^{\prime }\alpha \right)-\left(\pi_{k-1}-x_{i}^{\prime }\alpha \right)$$

φ(.) is the standard normal cumulative distribution function and K is the response category. Greene & Schlacther [34] greendeducted that since there is no meaningful conditional mean function and the marginal effects in the ordered probability function is not straightforward, the effect of changes in the explanatory variables on cell probabilities are normally considered.

These are given by

(8) $$\frac{\delta Prob\left(cellj\right)}{\delta x_{i}}=\left[\;\phi \left(\pi_{k-1}-x_{i}^{\prime }\alpha \right)-\left(\pi_{k}-x_{i}^{\prime }\alpha \right)\right]\times \alpha$$

Where ϕ(.) is the standard normal density function. The empirical model is then specified as:

(9) $${MTI}_{ik}=a+\gamma X_{i}+\varepsilon_{i}$$

Where MTI is the marginal soybean technology intensity, subscript i represents a household, while subscript k (k=0,1,2,3,) represents the four-pronged categorization of alternative dummy variables indicating (i) whether a household used all three soybean technologies (ISV, herbicides, fertilizer), (ii) whether a household used two soybean technologies, (iv) whether a household used one soybean technologies or (v) whether a household used no soybean technologies. $X_{i}$ represents household socioeconomic, location and plot level variables with γ being the parametric coefficient of X to be estimated.

## 3 Results and discussion

### 3.1 Summary statistics

The descriptive statistics of the sampled households (see Table 3) indicate that the average ages of male and female respondents were 47 and 48 years, respectively, suggesting that most farmers are in their productive years. The average household size was 8 persons for both male and female households. In terms of land cultivation, male farmers managed an average of 3.2 ha, while female farmers cultivated 2.4 ha. Both groups had 20 years of farming experience, demonstrating their extensive knowledge of crop production. Credit availability was notably low for both male and female farmers, reflecting a capital deficit during planting seasons. This lack of access to loans hinders their ability to expand production. Significant differences were observed in income and total household assets, with male farmers having higher levels than their female counterparts. Additionally, 35 % of male respondents and 27 % of female respondents were members of community-based organizations. These associations serve as vital sources of social capital, providing access to information on new technologies and support during emergencies. The higher membership rate among male farmers likely affords them greater access to technology and financial support compared to female farmers.

**Table 3:** Socio economic characteristics.

	Male	Female
Age (years)	47.400	48.000
Education of HH head	1.881	1.990
Household size	8.200	8.100
Farm Experience (years)	20.400	20.400
Membership of association (%)	35	27
Frequency of extension contact ()	4.840	5.060
Total credit (Naira)	1772.250	2047.000
Income (Naira)	41205.464	39495.738
Assets (Naira)	13638.971	12669.040
Tropical Livestock Unit size	0.837	0.979
Total land cultivated (ha)	3.200	2.400
Use of SSP fertilizer (%)	68.500	68
Use of herbicide (%)	0.17	0.15
Crop input cost (Naira)	23401.930	22609.580
Constrained by low soil fertility^a^	4.673	4.475
Constrained by high cost of inputs^a^	5.325	5.487
Constrained by pests and diseases	4.650	4.643
High oil content (%)	10	12.800
High fodder yield (%)	58	63
Less shattering (%)	38	35
Distance to seed market (km)	5.662	5.495
Distance to extension service (km)	7.138	6.992
Distance to chemical market (km)	1.446	1.363
Biu LGA (%)	32.800	32.000
Kwaya Kusar LGA (%)	25	29.500

a Perceived severity of constraints on a scale of 10, from zero (not constrained) to 10 (severely constrained), NGN: 360 NGN (Nigerian Naira) is equivalent to 1 USD at the survey time (see Table 3).

Both male and female farmers identified high input costs as a major constraint, with female farmers reporting a greater severity of this issue. Female farmers were more likely to adopt varieties for their higher oil content and fodder yield, while male farmers prioritized varieties with less pod shattering. The average distance to seed markets was around 6 km for both genders, a factor that may negatively impact the adoption and intensity of improved crop varieties due to limited seed availability.

### 3.2 Adoption of improved soybean management practices disaggregated by gender

The adoption of improved soybean management practices is documented in Table 4 below. Among the farmers surveyed, 72.38 %, have adopted improved soybean varieties (ISV). When broken down by gender, 75.25 % and 69.50 % of male and female farmers, respectively have adopted the improved varieties. This indicates that a slightly higher percentage of male farmers have adopted improved soybean varieties compared to female farmers, showing that male farmers are slightly more likely to adopt these varieties. Compared to other gender studies on the African continent, this difference of 5.75 % is small and shows some degree of parity in improved soybean adoption. For example, in Uganda, Bayiyana et al. [35] study on sweet potato varieties revealed that 60 % of male farmers were more likely to plant high-yielding varieties, while only 45 % of female farmers did as women faced more significant barriers to adoption due to limited access to information and planting materials. In another study by Jaleta et al. [36], the adoption rate of improved wheat varieties was 70 % among male farmers, compared to 55 % among female farmers in Ethiopia again with male farmers having 15 % more adoption than their female counterparts. In Malawi, Tufa et al. [37] revealed that female plot managers were 30 % less likely to adopt improved soybean varieties compared to male plot managers. All these other results show that crop interventions in Borno State by local and international partners were successful in not only ensuring a high adoption rate of improved soybean varieties but also in ensuring female farmers would not be left behind. This was a result of deliberate efforts by various intervention programs in the study area to integrate gender mainstreaming into their activities [2].

**Table 4:** Adoption of improved soybean management practices.

	Total		Male		Female	
Variable	Frequency (N = 800)	%	Frequency (N = 400)	%	Frequency (N = 400)	%
ISV	579	72.380	301	75.250	278	69.500
Use of fertilizer	546	68.250	274	68.500	272	68
Use of herbicide	124	15.500	66	16.500	58	14.500

About 68.25 % of the total sampled, reported using fertilizers. The results show that 68.5 % of male 68 % of female farmers used fertilizers on soybean. The negligible difference in fertilizer adoption between male and female farmers suggests that both genders equally recognize the importance of fertilizers in enhancing crop productivity and both have nearly equal access to fertilizers. When comparison is made to other gender studies in Africa it is obvious that there is comparative parity in access to inputs in our study area, Borno. A study on climate-smart agricultural (CSA) practices in sub-Saharan Africa found that 50 % of men and 35 % of women adopted modern chemical fertilizers, highlighting a more pronounced gender gap favoring male farmers [38] While in Ethiopia, Kebede [39] revealed that the adoption rate of chemical fertilizers showed a 10 % gap, with 40 % of male-headed households and 30 % of female-headed households adopting fertilizers. This gap persisted despite accounting for household characteristics and access to resources [39]. In Malawi and Tanzania, a study on improved seed and fertilizer use revealed that male plot managers were more likely to adopt fertilizers. In Malawi, 45 % of male plot managers growing maize used fertilizers compared to 35 % of female maize plot managers. In Tanzania, the rates were closer, with 40 % of male and 30 % of female maize plot managers using fertilizers [40].

The overall adoption rate for herbicides is lower among male and female farmers, with only 15.5 %, utilizing herbicides. Among male farmers, 16.5 %, use herbicides, while 14.5 %, of female farmers, also use herbicides. This low rate of herbicides might be due to either unavailability, high cost, or lack of knowledge on herbicide application*.* In comparison to other studies where herbicide use was reported to be higher in general, our result shows considerably less disparity in use of herbicides between male and female farmers. Farnworth et al. [41] found that while 35 % of male farmers adopted herbicides as part of conservation agriculture (CA) practice, in southern Africa only 25 % of female farmers did so. The study also noted that gender biases in extension services affected women's access to herbicides and other CA technologies. Similarly in Ghana, Kpienbaareh et al. [42] explored the drivers of herbicide use among smallholder farmers and found that 45 % of male farmers used herbicides compared to 35 % of female farmers.

The results from this study imply that the gender mainstreaming activities implemented by various soybean intervention projects and policymakers in Borno State have been effective. The high and relatively equitable adoption rates of improved soybean varieties, fertilizers, and herbicides among male and female farmers indicate that extension services and agricultural support have been accessible to both genders. This success suggests that efforts to address gender disparities in agricultural practices and inputs have made significant strides.

### 3.3 Intensity of adoption of improved soybean management practices

The adoption intensity of improved soybean management practices (ISMPs) among male and female farmers is recorded in Table 5. Among the total 800 farmers surveyed, 12.63 %, did not use any improved soybean management practices. When disaggregated by gender, 11.5 % of male and 13.75 % of female farmers did not adopt any ISMPs, indicating a slightly higher percentage of female farmers did not engage in improved practices compared to male farmers. For those who adopted at least one improved practice, 29.5 % of the total, used one ISMP. Both male and female farmers showed equal adoption rates for a single practice, 29.5 %, in each group. This parity suggests that when it comes to adopting at least one practice, there is no significant gender disparity. A more pronounced adoption is observed with two practices, where 47 %, adopted two ISMPs. Among them, 46.25 %of males and 47.75 % of females adopted two practices. The slight edge for female farmers indicates a marginally higher intensity of adoption among women for this category (see Table 5).

**Table 5:** Adoption intensity of ISMPs.

	Total		Male		Female	
Number of improved soybean management practices used	Frequency (N = 800)	%	Frequency (N = 400)	%	Frequency (N = 400)	%
0	101	12.630	46	11.500	55	13.750
1	236	29.500	118	29.500	118	29.500
2	376	47	185	46.250	191	47.750
3	87	10.880	51	12.750	36	9

**Table 6:** Determinants of multiple soybean management practices using multivariate probit model among male farmers.

Variables	Improved soybean varieties	Herbicide use	Fertilizer use
Age	−0.021∗∗∗ (0.009)	0.004 (0.010)	−0.006 (0.009)
Education (years)	0.083 (0.060)	0.034 (0.059)	0.065 (0.052)
Membership in Association (years)	−0.014 (0.021)	0.006 (0.022)	−0.010 (0.019)
Household size	−0.013 (0.017)	0.020 (0.019)	0.003 (0.017)
Farming experience	−0.003 (0.01)	−0.020∗∗ (0.01)	0.005 (0.009)
Tropical livestock unit	0.022 (0.037)	−0.052 (0.058)	−0.018 (0.032)
Number of extension visits	0.034 (0.024)	0.004 (0.007)	0.003 (0.007)
Years resident in the community	0.045∗∗∗ (0.006)	0.011 (0.007)	−0.001 (0.005)
Credit	0.047 (0.038)	0.006 (0.037)	0.01 (0.036)
Per capita income	1.24E-05∗∗∗ (2.77E-06)	−3.29E-06 (2.58E-06)	−4.78E-07 (1.23E-06)
Assets per capita	−3.16E-06 (2.45E-06)	2.04E-06 (2.99E-06)	−8.62E-07 (2.99E-06)
Farm size	0.105 (0.159)	0.989∗∗∗ (0.172)	1.638∗∗∗ (0.231)
Crop input cost (Naira)	−1.99E-06 (2.48E-06)	1.99E-05 (2.60E-06)	2.51E-05∗∗∗ (3.26E-06)
Distance to seed market (km)	0.095 (0.081)	−0.021 (0.086)	−0.038 (0.072)
Constrained by low soil fertility1	0.001 (0.021)	−0.010 (0.022)	−0.019 (0.019)
Constrained by high cost of inputs1	−0.012 (0.023)	0.040∗ (0.024)	−0.018 (0.020)
Constrained by pests and diseases1	0.034 (0.022)	−0.025 (0.024)	0.067∗∗∗ (0.020)
Distance to chemical market (km)	−0.157 (0.121)	0.066 (0.126)	0.093 (0.111)
Distance to tarmac (km)	0.052 (0.080)	−0.040 (0.089)	−0.154∗∗ (0.07)
Kwaya Kusar LGA	−0.408∗ (0.214)	0.192 (0.243)	0.008 (0.199)
Hawul LGA	0.498 (0.211)	0.029 (0.213)	0.338 (0.183)
Constant	−0.162 (0.510)	−3.15∗∗ (0.571)	−1.783 (0.469)

∗∗∗∗∗,∗ represent significance level at 1 %, 5 % and 10 %; Standard error clustered at village level.

The adoption of all three improved practices was less common, with only 10.88 %, adopting three ISMPs. Among these, 12.75 % of male and 9 % of female-headed households adopted all three practices, showing that male farmers were more likely to adopt the full suite of improved practices compared to female farmers. In a similar study by Meguza-Tembo et al. [43], they showed that approximately 40 % of farmers did not use any climate change adaptation strategy (CAS). About 17.94 % used only one strategy with 15.3 and 22.8 percent using 2 and 3 strategies respectively. This shows that while our adoption rate is generally high, the intensity of adoption for 3 strategies was significantly higher in Southern Malawi.

### 3.4 Determinants of improved soybean varieties adoption among male and female farmers

The results of the multivariate probit model assessing the factors influencing the adoption of improved soybean management practices among male and female farmers are shown in Table 6, Table 7, respectively. In comparing the adoption of improved soybean varieties between male (see Table 6) and female farmers (see Table 7), several key differences and similarities emerge, as supported by existing literature. For male and female farmers, the study observed a highly significant positive (P<1 %) relationship between the number of years a farmer has resided in their community and the adoption of improved soybean varieties This suggests that longer community tenure facilitates trust and credibility, which in turn encourages the adoption of new agricultural practices. This finding is corroborated by studies such as Okori et al. [44], who found that prolonged residence in a community in Malawi improved access to improved seeds through social networks and community seed banks. Similarly, Adebayo et al. [45] reported that years of residence and community integration in Benue State, Nigeria, were significant factors in the adoption of improved soybean technologies among male farmers. Aman and Okoyo [46] in Ethiopia, also noted that longer community tenure among smallholder farmers increased the likelihood of adopting improved wheat varieties. However, some studies, such as Shuaibu [47] in Kano State, Nigeria, suggest that the duration of residency might not be as critical for female farmers, with other factors like education and access to extension services playing more prominent roles.

Income was another significant factor influencing adoption. Generally, farmers with higher per capita income were more likely to adopt improved soybean varieties, as greater financial resources enabled them to invest in these potentially profitable technologies [48,49] Higher per capita income also allows for investment in high-quality seeds and necessary inputs [50,51]. However, Jaleta et al. [36] found that despite higher incomes, female farmers in Ethiopia were less likely to adopt improved wheat varieties due to social and institutional barriers, highlighting that income alone may not be sufficient for adoption among female farmers.

Pest and disease constraints also played a significant role. Both sets of farmers facing such constraints were more likely to adopt improved soybean varieties, perceiving them as solutions to enhance crop resilience [52,53]. This trend was similarly observed by Checco et al. [54] who reported that in the Global South, pest and disease resistance was a critical determinant in the adoption of improved varieties by female farmers. However, significant gender differences emerged in other areas. For instance, female farmers showed a significant negative relationship between the number of extension visits and the adoption of improved soybean varieties (significant at the 1 % probability level). This might reflect inadequacies in the extension services provided, which may not sufficiently address the specific needs and preferences of female farmers [55,56]. Conversely, extension visits were generally found to positively influence adoption rates among male farmers in other studies, particularly when these services were well-structured and effectively delivered [57]. Female farmers were more likely to reduce their adoption of improved soybean varieties in response to higher input costs, reflecting greater sensitivity to financial constraints [58,59]. This disparity is likely driven by female farmers having limited access to critical resources, which can be traced to historical and systemic inequalities in land ownership, financial capital, and agricultural support services [6]. In many societies, land tenure systems have traditionally favored male ownership, restricting women's ability to acquire, inherit, or use land as collateral for loans, thereby limiting their investment in improved agricultural technologies [60]. Additionally, financial constraints stemming from gender-biased credit systems and lower income-generating opportunities further reduce female farmers' ability to purchase inputs such as fertilizers, improved seeds, and herbicides [61].

### 3.5 Determinants of herbicide use among male and female farmers

For male farmers, the study as reported in Table 6 found that extensive farming experience negatively influences the adoption of herbicides (significant at the 5 % probability level). This suggests that seasoned male farmers may be more rooted in traditional farming methods and skeptical of adopting new practices like herbicide use due to perceived costs and potential risks. This finding is contrary to *apriori* expectation of farming experience increasing the likelihood of farmers adopting pesticide use. Contrary to male farmers, age plays a significant and positive role in the adoption of herbicides (significant at the 5 % probability level) by female farmers. As female farmers (see Table 7) advance in age, their inclination to adopt herbicides increases, likely due to accumulated experience and exposure to modern farming practices. This finding is supported by studies such as Igwe [62], who found that older female farmers in Ebonyi State, Nigeria, were more likely to adopt sustainable agricultural practices, including herbicide use. Rahman and Chidiebere [30] also reported a positive relationship between farming experience and pesticide use. However, studies of Kebede and Tariku [63]) found that older female farmers in Northern Ethiopia were less likely to adopt improved bread wheat varieties, indicating that the influence of age can vary depending on the context and specific agricultural technology.

Farm size was another significant factor for male farmers, with those with larger farms being more likely to adopt herbicides (significant at the 1 % probability level). This relationship is driven by the increased need for efficient weed management on larger plots of land, making herbicides a practical choice to maximize productivity. Moreover, high labour costs and drudgery has made manual weeding unattractive in the West Africa sub-region. Therefore, those with large farm size prefer to use the most cost-effective method if using herbicides on their farms. Similarly, Anang, and Amikuzuno [64] reported that farm size was positively related to pesticide use in rice production in northern Ghana. Thus, an increase in farm size increases the probability of the adoption of pesticides in rice production in the study area. Our finding is also consistent with those of Alabi et al. [65] who found a positive relation between agrochemical use and farm size in the Federal Capital Territory, Nigeria. From our study, we deduce that as farm size increases, farmers are unable to effectively control weeds manually without resorting to pesticide use. Hence an increase in farm size increases the likelihood of herbicide use in soybean production by farmers. In contrast, female farmers did not show a significant relationship between farm size and herbicide adoption probably due to high cost of herbicides which the female farmers could not afford. Yasinet al. [66], reported that farm size negatively influenced farmer's receptivity to citrus spray with pesticides in Pakistan.

Education played a notable role, though with a lower level of significance (at the 10 % probability level). Female farmers with more years of formal education were more likely to adopt herbicides, as education equips them with the knowledge and skills necessary to implement new agricultural practices effectively. This finding aligns with Etim and Ndaeyo [67], who found that higher education levels among female rice farmers in Akwa Ibom State, Nigeria, increased the likelihood of adopting climate-smart agricultural practices, including herbicide use. Oyinbo and Zibah [68] reported that educational level (P < 0.01) is among the socioeconomic variables significantly influencing herbicide utilization by maize farming households in northern Nigeria. However, Gyimah et al. [69] noted that in Sub-Saharan Africa, education alone did not always translate to higher adoption rates of climate-smart agriculture among female farmers, highlighting the importance of complementary factors like access to credit and market information.

Finally, distance to seed markets showed a significant negative relationship with herbicide adoption for female farmers (significant at the 5 % probability level). Female farmers residing closer to seed markets were more likely to adopt herbicides, likely due to easier access to agricultural inputs and reduced transportation costs. This finding is in line with studies of Kangogo et al. [70] who highlighted that farmers closer to urban centers in Malawi were more likely to adopt improved seeds and other agricultural technologies due to better market access. However, this factor did not emerge as significant for male farmers in the context of herbicide adoption.

### 3.6 Determinants of fertilizer use among male and female farmers

When comparing the factors influencing fertilizer use between male (Table 6) and female farmers (Table 7), distinct differences and similarities emerge, as supported by various studies. For male farmers, farm size plays a significant role in fertilizer adoption. The highly significant positive coefficient of 1.64 (significant at the 1 % probability level) indicates that larger farm sizes strongly correlate with increased fertilizer use because large-scale farmers are likely to have the resources to afford fertilizer costs. Larger-scale farming operations, which typically aim for higher yields, are more likely to adopt fertilizers to enhance soil fertility and crop output. This trend is consistent with findings of Paudel et al. [71] in Nepal, where larger farm size was positively associated with fertilizer adoption, and Zhang et al. [72] in China, where economic levels and farm size were key drivers of fertilizer consumption. In contrast, for female farmers, farm size also significantly influenced fertilizer use (significant at the 1 % probability level), but the relationship reflects the financial resources available to those with larger landholdings. Female farmers with larger farms are more likely to invest in fertilizers to optimize crop yields, as observed in studies of Dossah & Mohammed [73] in Nigeria and Mensah, Villamor, & Vlek [74] in Ghana.

For female farmers, input costs have a negative effect on fertilizer application (significant at the 1 % probability level). This is because the financial burden imposed by rising input costs acts as a barrier to fertilizer use. Female farmers facing high input costs are more likely to economize and potentially reduce fertilizer use, as indicated by the significant negative coefficient. This is similar to Balasha et al. [75] who reported that credit constraints and rising input costs acted as barriers to the use of crucial inputs like fertilizers by women farmers in the Democratic Republic of Congo. Ayodeji et al. [76] also reported that in Nigeria credit constraint limited female farmers from accessing inputs like fertilizer.

The influence of pests and diseases on fertilizer adoption presents another interesting comparison. For male farmers, the positive coefficient of 0.067 (significant at the 1 % probability level) indicates that challenges related to pests and diseases drive fertilizer adoption as a strategy to strengthen crop resistance and overall plant health. This trend is consistent with Praveen [77] in India, who noted that the presence of high-yielding variety (HYV) crops, often susceptible to pests and diseases, necessitates additional fertilizer to support crop productivity. For female farmers, the influence of pests and diseases also positively impacts fertilizer use (significant at the 10 % probability level), as these constraints prompt a strategic approach to enhance crop resilience. Studies such as Bhandari et al. [78] in Nepal and Igwe [62] in Nigeria similarly highlight that female farmers use fertilizers as part of an integrated pest management strategy to bolster crop health.

Proximity to infrastructure reveals differing impacts on fertilizer use between genders. For male farmers, the negative coefficient of −0.154 (significant at the 5 % probability level) suggests that those living farther away from tarmac roads are less likely to adopt fertilizers. This is logical as it indicates that the farther away a farmer is from access to good roads the less likely they have access to input market and extension offices. This finding is supported by Adepoju and Salman [79] in Nigeria, who observed that better access to roads and infrastructure often correlates with increased agricultural technology adoption due to increases in access to extension services.

### 3.7 Assessment of intensity of adoption of improved management practices of male and female farmers using the ordered probit model

The findings from the ordered probit regression on the intensity of adoption of improved soybean management practices among male and female farmers are presented in Table 8, Table 9 respectively. The chi-squared statistics from the ordered probit model in both results is statistically significant, suggesting that the model is of good fit. When comparing the intensity of adoption of improved soybean management practices between male and female farmers, several key differences and similarities emerge, as highlighted by the ordered probit regression analysis.

**Table 7:** **Determinants of multiple soybean management practices using multivariate probit model among** female farmers.

Variable	Improved soybean varieties	Herbicide use	Fertilizer use
Age	−0.040∗∗∗ (0.011)	0.019 (0.0100)	−0.009 (0.009)
Education (years)	0.074 (0.055)	0.077 (0.051)	0.046 (0.047)
Membership in Association (years)	0.012 (0.021)	−0.032 (0.025)	−0.015 (0.016)
Household size	−0.021 (0.023)	0.022 (0.022)	0.018 (0.018)
Farming experience	−0.002 (0.012)	−0.021∗∗∗ (0.010)	0.005 (0.009)
Tropical livestock unit	−0.003 (0.030)	0.025 (0.030)	0.016 (0.032)
Number of extension visits	−0.016∗∗∗ (0.007)	0.004 (0.007)	−0.001 (0.006)
Years resident in the community	0.070∗∗∗ (0.007)	−0.002 (0.007)	0.005 (0.006)
Log credit	0.007 (0.039)	−0.014 (0.040)	0.020 (0.031)
Per capita income	1.47E-05∗∗∗ (2.96E-06)	−1.54E-06 (1.81E-06)	7.47E-07 (1.57E-06)
Assets per capita	6.28E-06 (5.09E-06)	−2.09E-06 (4.78E-06)	1.00E-06 (4.05E-06)
Farm size	−0.021 (0.166)	0.958∗∗∗ (0.182)	2.081∗∗∗ (0.248)
Crop input cost (Naira)	−5.96E-06∗∗∗ (2.75E-06)	1.92E-05∗∗∗ (2.85E-06)	2.71E-05∗∗∗ (3.63E-06)
Distance to seed market (km)	0.140 (0.091)	−0.206∗∗ (0.089)	−0.112 (0.073)
Constrained by low soil fertility1	−0.001 (0.023)	−0.010 (0.025)	0.016 (0.0200)
Constrained by high cost of inputs1	−0.036 (0.025)	−0.038 (0.024)	−0.057∗∗∗ (0.021)
Constrained by pests and diseases1	−0.002 (0.024)	0.008 (0.023)	0.033 (0.020)
Distance to chemical market (km)	0.227 (0.151)	0.119 (0.132)	0.114 (0.115)
Distance to tarmac (km)	0.331∗∗∗ (0.103)	0.027 (0.095)	0.099 (0.178)
Kwaya Kusar LGA	−0.311 (0.218)	−0.03 (0.224)	−0.192 (0.192)
Hawul LGA	0.795∗∗∗ (0.237)	−0.305 (0.231)	−0.078 (0.179)
Constant	0.106 (0.553)	−2.973∗∗∗ (0.565)	−1.988 (0.507)

∗∗∗,∗∗,∗ represent significance level at 1 %, 5 % and 10 %; Standard error clustered at village level.

**Table 8:** **Determinants of intensity of adoption of improved management practices of** male farmers **using the ordered probit model**.

Variable	Coefficient	Standard error	t-value
Age	−0.009∗∗∗	0.003	−2.53
Education	0.007	0.071	0.10
Membership in Association	−0.023	0.048	−0.49
Household size	−0.089	0.068	−1.30
Farming experience	−0.086	0.065	−1.32
Tropical livestock unit	−0.011	0.071	−0.17
Number of extension visits	0.029	0.043	0.67
Years resident in the community	0.523∗∗∗	0.060	8.62
Credit	0.023	0.016	1.39
Per capita income	0.260∗∗∗	0.081	3.21
Assets per capita	−0.023	0.048	−0.47
Farm size	0.051	0.057	0.91
Crop input cost (Naira)	0.057	0.068	0.84
Distance to seed market (km)	0.309∗∗	0.130	2.37
Constrained by low soil fertility1	−0.006	0.010	−0.59
Constrained by high cost of inputs1	−0.012	0.010	−1.24
Constrained by pests and diseases1	−0.019∗	0.010	−1.84
Distance to chemical market (km)	0.052	0.058	0.89
Distance to tarmac (km)	0.328∗∗∗	0.114	2.86
Kwaya Kusar LGA	−0.202∗	0.104	−1.94
Hawul LGA	0.170∗	0.094	1.80
Cut1	4.4361	1.2655	
Cut2	5.5153	1.2702	
Cut3	7.0700	1.2757	
LR chi2(21) = 141.84			
Prob > chi2 = 0.0000			
Pseudo R2 = 0.072			

∗∗∗∗∗,∗ represent significance level at 1 %, 5 % and 10 %; Standard error clustered at village level.

**Table 9:** **Determinants of intensity of adoption of improved management practices of** female farmers **using the ordered probit model**.

Variable	Coefficient	Standard err	t-value
Age	−0.009∗∗∗	0.003	−2.54
Education	0.008	0.071	0.11
Membership in Association	−0.023	0.048	−0.47
Household size	−0.09	0.068	−1.32
Farming experience	−0.086	0.065	−1.32
Tropical livestock unit	−0.012	0.071	−0.18
Number of extension visits	0.029	0.043	0.67
Years resident in the community	0.523	0.46	1.62
Credit	0.023	0.016	1.39
Per capita income	0.26	0.181	1.2
Assets per capita	0.335	0.317	0.85
Farm size	0.051	0.057	0.89
Crop input cost (Naira)	−0.274∗∗	0.117	−2.33
Distance to seed market (km)	0.312∗∗	0.13	2.39
Constrained by low soil fertility1	−0.006	0.01	−0.59
Constrained by high cost of inputs1	−0.012	0.01	−1.24
Constrained by pests and diseases1	0.019∗	0.01	1.84
Distance to chemical market (km)	0.052	0.058	0.91
Distance to tarmac (km)	0.003	0.014	0.28
Kwaya Kusar LGA	−0.200∗	0.104	−1.92
Hawul LGA	0.17	0.194	0.8
Cut1	4.5052	1.2901	
Cut2	5.5844	1.2947	
Cut3	7.1393	1.3003	
LR chi2(21)	141.92		
Prob > chi2	0.0000		
Pseudo R2	0.0729		

∗∗∗,∗∗,∗ represent significance level at 1 %, 5 % and 10 %; Standard error clustered at village level.

For both male and female farmers, a notable negative coefficient for age suggests that as male and female farmers age, their likelihood of adopting a higher number of improved soybean management practices decreases. This finding implies that younger farmers are more likely toward modern agricultural practices. Similar trends are observed in the literature, with Maguza-Tembo et al. [43] reporting that in Southern Malawi, older farmers were less likely to adopt multiple climate change adaptation strategies, and Piedra-Bonilla et al. [80] noting that younger farmers in Brazil were more likely to adopt diversified crop management practices. Results indicate that older female farmers are less likely to adopt multiple improved soybean management practices. This trend suggests a preference for traditional farming methods among older women, as they are more likely to protect norms and traditions. Damisa & Yohanna [81] found similar results in Nigeria, where older female farmers were less likely to participate in farm management decision-making processes, including the adoption of modern practices.

For male farmers, a positive and significant coefficient for years of residence in the community suggests that the longer a male farmer has lived in a community, the more likely he is to adopt a greater number of improved soybean management practices. This could be due to increased familiarity with local agricultural dynamics and stronger community networks. Maguza-Tembo et al. [43] and Piedra-Bonilla et al. [80] also found that longer community residence positively influenced the adoption of multiple agricultural practices in Malawi and Brazil, respectively. In contrast, the analysis does not indicate a significant relationship between years of residence in the community and the intensity of adoption of improved practices among female farmers. For male farmers, higher per capita income is positively and significantly associated with the adoption of more improved soybean management practices, indicating that financial resources play a crucial role in facilitating the adoption of modern agricultural techniques. This finding aligns with Alemayehu et al. [82] in Ethiopia and Maguza-Tembo et al. [28] in Malawi, where higher income levels were associated with the adoption of multiple improved agricultural practices.

On the other hand, female farmers are more sensitive to high input costs, with a negative coefficient indicating that perceptions of high input costs decrease the likelihood of adopting more improved practices. This finding highlights the importance of addressing affordability barriers to encourage broader adoption among women. Maguza-Tembo et al. [28] and Suraningsih et al. [83] support this, noting that high input costs are a significant barrier to adoption among female farmers in Malawi and Indonesia.

Proximity to tarmac roads is positively associated with the intensity of adoption among male farmers, suggesting that better access to transportation infrastructure facilitates the adoption of agricultural innovations. Maguza-Tembo et al. [28] also found that better access to roads positively influenced the adoption of multiple climate adaptation strategies in Malawi. For female farmers, proximity to seed markets is a significant factor, with those closer to markets being more likely to adopt a higher number of improved practices. This suggests that access to resources, including transportation assets, is a critical constraint for women. Maguza-Tembo et al. [28] and Malhan & Rao [84] also found that proximity to markets positively influenced the adoption of agricultural practices among female farmers in Malawi and India. Interestingly, the influence of pests and diseases on adoption differs between genders. For male farmers, the presence of pests and diseases negatively impacts the likelihood of adopting improved practices, suggesting that pest and disease pressures may deter adoption. This contrasts with female farmers, where pest and disease constraints are positively associated with the adoption of more improved practices. Female farmers may view these challenges as a prompt to adopt more robust management practices to safeguard their crops. Maguza-Tembo et al. [28] found similar gender-specific responses in Malawi, where pest and disease pressures influenced the adoption of climate-smart agricultural practices differently between men and women. Finally, regional differences also play a role. Male farmers residing in Hawul LGA are more likely to adopt a higher number of improved practices, while those in Kwaya Kusar LGA are less likely to do so. Conversely, female farmers in Hawul LGA show lower adoption rates, possibly due to limited access to resources. Alemayehu et al. [82] found that regional differences within Ethiopia significantly influenced the adoption of agricultural practices, with areas having better infrastructure and support systems showing higher adoption rates.

## 4 Conclusion

This study examined gender disparities in the adoption and intensity of improved soybean management practices in Borno State, Northeast Nigeria, using sex-disaggregated data from 800 farming households. The findings suggest that while both male and female farmers adopt improved soybean varieties, fertilizer, and herbicides, the intensity of adoption varies due to differences in socioeconomic constraints. The results indicate that farm size and input costs are critical determinants of adoption for both genders, but female farmers face additional barriers, including limited access to credit, extension services, and proximity to input markets.

The study also reveals that male farmers are more likely to adopt improved practices when they have higher per capita income, more years of residence in their communities, and access to extension services. In contrast, female farmers are more influenced by factors such as crop input costs, distance to input markets, and the number of extension visits received. The role of infrastructure is particularly important, as residing in certain local government areas (LGAs), such as Kwaya Kusar and Hawul, significantly impacts the intensity of adoption among both male and female farmers.

These findings suggest that the local extension service should expand its reach to improve adoption rates among all farmers. The introduction of targeted subsidies by policymakers at the federal level, for female farmers can help alleviate financial constraints and encourage greater adoption of improved soybean management practices. Expanding financial support mechanisms, such as credit programs tailored to smallholder farmers, will enable both male and female farmers to invest in essential inputs and scale up their production. Additionally, improving road networks and proximity to input markets by policymakers at the state level will reduce logistical challenges, making it easier for farmers to access the necessary resources for soybean cultivation.

## CRediT authorship contribution statement

**Amadu Yaya Kamara:** Writing – review & editing, Writing – original draft, Visualization, Methodology, Investigation, Formal analysis, Data curation, Conceptualization. **Lucy Sahbong Kamsang:** Validation, Supervision, Project administration, Methodology, Investigation. **Amina Mustapha:** Writing – review & editing, Validation, Supervision, Conceptualization. **Alpha Yaya Kamara:** Writing – review & editing, Resources, Project administration, Funding acquisition, Conceptualization. **Adetomiwa Kolapo:** Writing – review & editing, Writing – original draft. **Nkeki Kamai:** Supervision, Resources, Project administration.

## Availability of data and materials

The authors declare that the data and file supporting the findings of this study are available on request.

## Funding

The Research was funded by the 10.13039/100000865Bill and Melinda Gates Foundation through the project Putting Nitrogen Fixation into Use for Smallholder farmers in Africa with Grant no. OPP1020032.

## Declaration of competing interest

The authors affirm that there are no conflicts of interest related to this study. They have no financial, personal, or professional affiliations that could be perceived as influencing the research outcomes or interpretations. All findings and conclusions presented in this paper are the result of independent academic research, conducted without any outside influence from funding bodies, organizations, or stakeholders that might benefit from the study's results. The authors have ensured full transparency throughout the research process, affirming their commitment to the integrity of the research and its contribution to the academic community.

## Data Availability

Data will be made available on request.
